# The Combination Effect of Aspalathin and Phenylpyruvic Acid-2-*O*-β-d-glucoside from Rooibos against Hyperglycemia-Induced Cardiac Damage: An In Vitro Study

**DOI:** 10.3390/nu12041151

**Published:** 2020-04-20

**Authors:** Phiwayinkosi V. Dludla, Christo J. F. Muller, Johan Louw, Sithandiwe E. Mazibuko-Mbeje, Luca Tiano, Sonia Silvestri, Patrick Orlando, Fabio Marcheggiani, Ilenia Cirilli, Nireshni Chellan, Samira Ghoor, Bongani B. Nkambule, M. Faadiel Essop, Barbara Huisamen, Rabia Johnson

**Affiliations:** 1 Biomedical Research and Innovation Platform, South African Medical Research Council, Tygerberg 7505, South Africa; christo.muller@mrc.ac.za (C.J.F.M.); johan.louw@mrc.ac.za (J.L.); sithandiwe.mazibukombeje@gmail.com (S.E.M.-M.); nireshni.chellan@mrc.ac.za (N.C.); samira.ghoor@mrc.ac.za (S.G.); bh3@sun.ac.za (B.H.); rabia.johnson@mrc.ac.za (R.J.); 2 Department of Life and Environmental Sciences, Polytechnic University of Marche, 60131 Ancona, Italy; l.tiano@univpm.it (L.T.); s.silvestri@univpm.it (S.S.); p.orlando@univpm.it (P.O.); f.marcheggiani@univpm.it (F.M.); ilenia.cirilli@unicam.it (I.C.); 3 Division of Medical Physiology, Faculty of Health Sciences, Stellenbosch University, Private Bag X1, Tygerberg 7505, South Africa; 4 Department of Biochemistry and Microbiology, University of Zululand, KwaDlangezwa 3880, South Africa; 5 School of Pharmacy, University of Camerino, 62032 Camerino, Italy; 6 School of Laboratory Medicine and Medical Sciences, College of Health Sciences, University of KwaZulu-Natal, Durban 4000, South Africa; nkambuleb@ukzn.ac.za; 7 Centre for Cardio-metabolic Research in Africa, Department of Physiological Sciences, Stellenbosch University, Stellenbosch 7600, South Africa; mfessop@sun.ac.za

**Keywords:** rooibos, aspalathin, phenylpropenoic acid glucoside, diabetes, hyperglycemia, oxidative stress, apoptosis

## Abstract

Recent evidence shows that rooibos compounds, aspalathin and phenylpyruvic acid-2-*O*-β-d-glucoside (PPAG), can independently protect cardiomyocytes from hyperglycemia-related reactive oxygen species (ROS). While aspalathin shows more potency by enhancing intracellular antioxidant defenses, PPAG acts more as an anti-apoptotic agent. Thus, to further understand the protective capabilities of these compounds against hyperglycemia-induced cardiac damage, their combinatory effect was investigated and compared to metformin. An in vitro model of H9c2 cardiomyocytes exposed to chronic glucose concentrations was employed to study the impact of such compounds on hyperglycemia-induced damage. Here, high glucose exposure impaired myocardial substrate utilization by abnormally enhancing free fatty acid oxidation while concomitantly suppressing glucose oxidation. This was paralleled by altered expression of genes involved in energy metabolism including acetyl-CoA carboxylase (ACC), 5′ AMP-activated protein kinase (AMPK), and peroxisome proliferator-activated receptor-alpha (PPARα). The combination treatment improved myocardial substrate metabolism, maintained mitochondrial membrane potential, and attenuated various markers for oxidative stress including nicotinamide adenine dinucleotide phosphate (NADPH) oxidase activity and glutathione content. It also showed a much-improved effect by ameliorating DNA damage when compared to metformin. The current study demonstrates that rooibos compounds offer unique cardioprotective properties against hyperglycemia-induced and potentially against diabetes-induced cardiac damage. These data also support further exploration of rooibos compounds to better assess the cardioprotective effects of different bioactive compound combinations.

## 1. Introduction

The escalating diabetes epidemic is a major contributor to the rising burden of noncommunicable diseases [1], greatly impacting the quality of life of those affected [2]. Diabetic patients present with an increased cluster of metabolic abnormalities that may include macrovascular complications such as coronary artery disease, stroke, and peripheral vascular disease [3]. In actual fact, cardiovascular complications remain the leading cause of death in diabetic patients [4]. Although pathophysiological mechanisms explaining deteriorated cardiac function in a diabetic state are diverse, a chronic hyperglycemic state is implicated in the initiation and propagation of these abnormalities. For example, persistently elevated glycemic levels are linked with impaired myocardial substrate metabolism as characterized by excess storage and usage of free fatty acids (FFAs) [5,6]. Although FFAs are the preferred substrate for energy generation in the myocardium, their excess utilization can impede the efficiency of the electron transport chain and raise mitochondrial membrane potential under hyperglycemic conditions [6]. This state can lead to excess production of reactive oxygen species (ROS) occurring together with depletion of intracellular antioxidant defenses which could lead to accelerated cardiac oxidative damage [7]. For this reason, several naturally derived products such as rooibos (*Aspalathus linearis*), with robust antioxidant properties, are increasingly explored for their ameliorative effects against hyperglycemia and its associated complications [8,9,10].

As recently reviewed [11,12,13], available literature strongly supports the ameliorative potential of rooibos tea against diabetes-associated complications, including cardiovascular anomalies [14,15]. Consistently, bioactive compounds present in rooibos like aspalathin and phenylpyruvic acid-2-*O*-β-d-glucoside (PPAG) can alleviate hyperglycemia-induced oxidative stress [16]. The therapeutic mechanisms associated with the effectiveness of these compounds have been partially explained, with aspalathin demonstrating a strong capacity to enhance endogenous antioxidants leading to reduced oxidative stress [17,18]. On the other hand, PPAG lacks antioxidant-enhancing capability but can blunt hyperglycemia-induced cellular apoptosis [19,20,21]. In fact, evidence from cultured H9c2 cardiomyocytes supports the combination use of metformin and PPAG in blunting high glucose-induced oxidative stress than the use of this rooibos compound as a monotherapy [19]. Such work, together with evidence demonstrating the cardioprotective potential of a fermented rooibos extract containing relatively high levels of both compounds [22,23], necessitates further exploration of the combination effect of aspalathin and PPAG to ameliorate hyperglycemia-induced oxidative damage in cultured cardiomyocytes.

## 2. Materials and Methods

### 2.1. Chemicals and Reagents

Aspalathin (ca. 98%, batch SZI-356-54) and PPAG (99% purity; batch: MC1(2)-248–91D) were obtained from High Force Research (Durham, UK). Embryonic ventricular rat heart derived H9c2 cardiomyoblasts (CRL-1446) were from American Type Culture Collection (Manassas, VA, USA); 14C-palmitate and 2-deoxy-[^3^H]-D-glucose were from American Radiolabeled Chemicals (St. Louis, MI, USA); fetal bovine serum (FBS) and horse serum were from Biochrom (Berlin, Germany); Dulbecco’s Modified Eagle’s Medium (DMEM), Dulbecco’s Phosphate Buffered Saline (DPBS), and Hank’s Balanced Salt Solution (HBSS) were from Lonza (Basel, Switzerland); 2′, 7′-dichlorofluorescein diacetate (DCFH-DA) fluorescent dye was from Cell Biolabs Inc. (San Diego, CA, USA); and 7-amino-4chloromethylcoumarin (CellTracker Blue CMAC) and TRIzol reagent were from Invitrogen Corp. (Carlsbad, CA, USA). All other chemicals, including 5,5′6,6′-tetrachloro-1,1′,3,3-tetraethylbenzimidazolyl-carbocyanine iodide (JC-1) and propidium iodide stain were obtained from Sigma-Aldrich (St. Louis, MI, USA).

### 2.2. Cell Culture and Treatment Conditions

H9c2 cardiomyoblasts were cultured in DMEM supplemented with 10% FBS, 100 μg/mL penicillin, and 100 μg/mL streptomycin overnight under standard tissue culture conditions (37 °C in humidified air and 5% CO_2_). Depending on the assay performed, cells were seeded in either 6-, 24-, or 96-well plate at a seeding density of 2 × 10^4^, 1 × 10^4^, or 0.8 × 10^4^, respectively. Confluent H9c2 cardiomyoblasts were differentiated into adult cardiomyocytes by replacing normal growth media with differentiation media consisting of DMEM containing 10 nM all-trans-retinoic acid and 1% horse serum for six days, as previously described [18]. Differentiated H9c2 cardiomyocytes were exposed to 33 mM glucose for 48 h prior to treatment with a combination of aspalathin and PPAG both at 1 μM for an additional 6 h. Metformin, as a well-known hypoglycemic drug, was used as a positive control at 1 μM also for 6 h. Cells exposed to 5.5 and 33 mM glucose concentrations served as controls for normoglycemic and hyperglycemic states. Consistent with our previous studies [17,18], the concentrations of glucose (5.5 and 33 mM) were prepared and added using glucose free DMEM powder from Sigma-Aldrich. These glucose solutions were filter sterilized before use in cell culture procedures. All selected doses for treatment compounds and experimental controls were based on previously published studies [17,18,19,23]. Cells exposed to 33 mM mannitol were used to rule out the effect of osmolarity, as previously reported [18,19].

### 2.3. FFA and Glucose Oxidation Assays

FFA and glucose oxidation assays were determined using previously described methods [18,24]. Briefly, treated H9c2 cardiomyocytes were cultured in DMEM containing either 5 μCi/mL of PAL-D-[^14^C (U)] or 0.5 μCi/mL of 2-deoxy-[^3^H]-D-glucose for 6 h in 6-well tissue culture plates, covered with filter paper moistened with 0.1 M NaOH. Thereafter, the filter paper was removed and the absorbed ^14^CO_2_ as a product of PAL-D-[^14^C (U)] and 2-deoxy-[^3^H]-D-glucose was detected by liquid scintillation for detection of FFA and glucose oxidation, using 2220 CA, Packard Tri-Carb series, PerkinElmer, IL, USA. Data are expressed relative to the control in arbitrary units.

### 2.4. Determination of Mitochondrial Membrane Potential (ΔΨm)

JC-1 stain was used to assess mitochondrial membrane depolarization in treated H9c2 cardiomyocytes, as previously described [25]. Briefly, treated H9c2 cardiomyocytes were washed with warm DPBS before staining with 2 μM JC-1 solution in DMEM without phenol red before incubation at standard tissue culture conditions for 30 min in the dark. Thereafter, cardiomyocytes were washed with warm DPBS and the fluorescence was measured at excitation of 485 ± 20 nm and dual emission of 530 ± 25 nm and 590 ± 35 nm, using a BioTek FLx800 plate reader (BioTek Instruments Inc., Winooski, VT, USA) equipped with a Gen 5 software (BioTek Instruments Inc., Winooski, VT, USA). Fluorescent photomicrographs were also taken at 100x magnification using a Nikon Eclipse Ti inverted microscope (Nikon, Tokyo, Japan), equipped with a NIS-Elements imaging software. The results for mitochondrial membrane potential were expressed as relevant fluorescent intensity (RFI) of red (depolarized)/green (healthy) fluorescent intensity of cardiomyocytes.

### 2.5. mRNA Expression Analysis

Total RNA was extracted from treated H9c2 cardiomyocytes using TRIzol reagent, while quantitative real time-PCR was performed on an ABI 7500 Instrument (Applied Biosystems, Foster City, CA, USA), as previously discussed [17]. Taqman gene expression assays used included Acetyl-CoA carboxylase (ACC; Rn00573474_m1), 5′ AMP-activated protein kinase (AMPK; Rn00576935_m1), glucose transporter four (GLUT4; Rn00562597_m1), and peroxisome proliferator-activated receptor alpha (PPARα; Rn00566193_m1). The quantitative RT-PCR conditions were as follows: 50 °C for 1 min and 95 for 10 min, followed by 40 cycles of 95 °C for 15 s and 60 °C for 30 s. Gene expression data were normalized to hypoxanthine-guanine phosphoribosyltransferase (HPRT; Rn01527840_m1).

### 2.6. Oxidative Stress Assessment

Intracellular ROS was detected using DCFH-DA fluorescent dye, per manufacturer’s instructions (Cell Biolabs Inc., San Diego, CA, USA). Briefly, final solution of 1 µM DCFH-DA was prepared in HBSS and added to cultured cardiomyocytes in 96-well plates before incubation under standard tissue culture conditions. Alternatively, an already described lucigenin-based method was used to determine NADPH oxidase (Nox) activity from cell lysates of treated cells [26,27]. Total glutathione (GSH) content was determined using 7-amino-4chloromethylcoumarin, as previously described [18]. Briefly, treated cells were exposed to 2.5 M CellTracker solution before incubation at 37 °C for 30 min. DCFH-DA (Ex/Em: 485 ± 20/ 528 ± 20 nm) and GSH (Ex/Em: 360 ± 20/ 460 ± 40 nm) fluorescent intensity, as well as luminescence to quantify Nox activity was measured using a BioTek FLx800 plate reader equipped with a Gen 5 software.

### 2.7. DNA Damage Assessment

DNA nicks were detected by labeling cells with DeadEnd Fluorometric Terminal deoxynucleotidyl transferase (TdT) dUTP (TUNEL) assay kit (Promega Corp., Madison, WI, USA), as previously described [28]. Direct visualization of green fluorescent staining was used to detect TUNEL-positive nuclei. The apoptotic rate was calculated as the average number of positive nuclei that overlapped with the TUNEL stain in non-overlapping fields of 1 mm^2^ under 100× magnification field, using a Nikon Eclipse Ti inverted fluorescent microscope equipped with a NIS Elements imaging software (Tokyo, Japan).

### 2.8. Statistical Analysis

Data were expressed as the mean ± SEM. Results were expressed as the mean of three independent biological experiments with each experiment containing at least three technical replicates. GraphPad Prism software version 6.0 (GraphPad Software, Inc., La Jolla, CA, USA) was used to analyze data. Comparisons between groups were performed using one-way multivariate ANOVA followed by a Tukey post hoc test and Student *t*-test where appropriate. A *p*-value of ≤ 0.05 was considered statistically significant.

## 3. Results

### 3.1. The Combination Effect of Aspalathin and PPAG on Myocardial Substrate Metabolism

**Figure 1:**
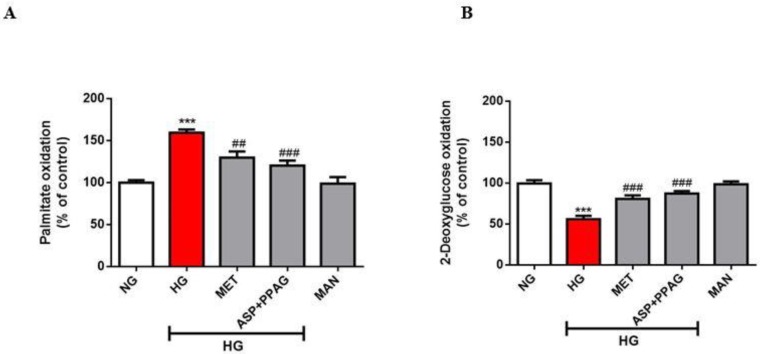
The combination effect of aspalathin (ASP) and phenylpyruvic acid-2-*O*-β-d-glucoside (PPAG) in comparison to metformin (MET) in ameliorating impaired myocardial substrate metabolism, as measured using palmitate (**A**) and 2-deoxyglucose (**B**) oxidation. H9c2 cardiomyocytes were exposed to 33 mM glucose (HG) for 48 h before treatment with a combination of ASP and PPAG, as well as MET, at a dose 1 μM for 6 h. Mannitol (MAN) at a concentration of 33 mM was used to rule out the effect of osmolarity. Results are expressed as the mean ± SEM of three independent experiments relative to the normal glucose (NG) control (5.5 mM). *** *p* < 0.001 versus NG control; ## *p* < 0.01 and ### *p* < 0.001 versus HG control.

Abnormally enhanced oxidation of FFAs accompanied by that of repressed glucose is a key feature of a diabetic heart that signals impaired myocardial substrate metabolism. In agreement, the current results showed that exposure of H9c2 cardiomyocytes to high glucose concentrations impaired substrate metabolism by raising FFA oxidation by 60% (*p* ˂ 0.001) and suppressing glucose oxidation by 44%; (*p* ˂ 0.001), when compared to the untreated control (Figure 1A,B). Treatment with the combination of aspalathin and PPAG could ameliorate this aberration by decreasing FFA oxidation by 44% (*p* ˂ 0.001) and improving glucose oxidation by 25% (*p* ˂ 0.001), when compared to the untreated high glucose control (Figure 1A,B). Consistent with the previous results for the use of each compound as a monotherapy [18,19], the combination effect of aspalathin and PPAG showed comparable efficacy to that of metformin in terms of modulating FFA and glucose oxidation.

### 3.2. The Combination Effect of Aspalathin and PPAG on Altered Mitochondrial Membrane Potential

**Figure 2:**
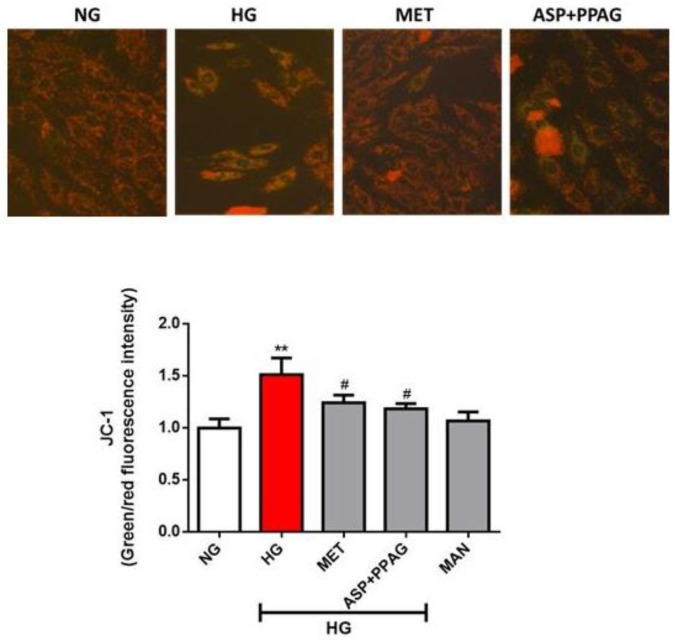
The combination effect of aspalathin (ASP) and phenylpyruvic acid-2-*O*-β-d-glucoside (PPAG) in comparison to metformin (MET) in improving mitochondrial membrane potential. H9c2 cardiomyocytes were exposed to 33 mM glucose (HG) for 48 h before treatment with a combination of ASP and PPAG, as well as MET, at a dose 1 μM for 6 h. Mannitol (MAN) at a concentration of 33 mM was used to rule out the effect of osmolarity. Results are expressed as the mean ± SEM of three independent experiments relative to the normal glucose (NG) control (5.5 mM). ** *p* < 0.01 versus NG control; # *p* < 0.05 versus HG control. Supporting images of JC-1 stained (green/red fluorescence) cardiomyocytes demonstrate an apparent change in morphology and loss in cell numbers for cells exposed to high glucose concentrations when compared to treated cells and controls.

Modifications in myocardial substrate metabolism are usually accompanied by altered mitochondrial membrane potential under hyperglycemic conditions. Chronic exposure of H9c2 cardiomyocytes to high glucose concentrations significantly elevated mitochondrial membrane potential (1.51 RFI; *p* ˂ 0.01), whereas treatment with the combination of aspalathin and PPAG (1.25 RFI; *p* ˂ 0.05) showed similar effect to metformin (1.05 RFI; *p* ˂ 0.05) in ameliorating this consequence (Figure 2). Supporting images of JC-1 stained cardiomyocytes demonstrate an apparent change in morphology and loss in cell numbers for cells exposed to high glucose concentrations when compared to treated cells and controls (Figure 2). Overall, these results are consistent with previous data demonstrating that both aspalathin and PPAG show comparative effects to metformin in ameliorating impaired mitochondrial membrane potential [18,19].

### 3.3. The Combination Effect of Aspalathin and PPAG on the Modulation of Genes Involved in Energy Metabolism

**Figure 3:**
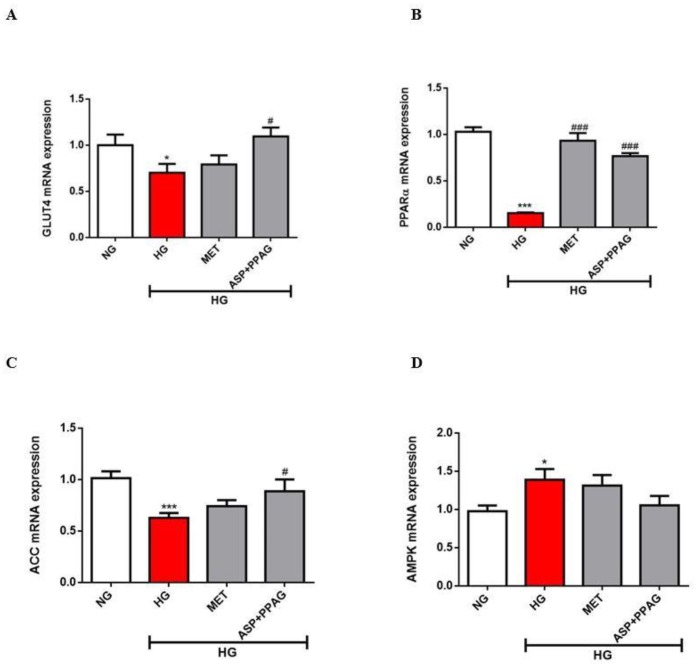
The combination effect of aspalathin (ASP) and phenylpyruvic acid-2-*O*-β-d-glucoside (PPAG) in comparison to metformin (MET) on the modulation of genes involved energy metabolism. Panels depict mRNA expression for (**A**) glucose transporter 4 (GLUT4, (**B**) peroxisome proliferator-activated receptor-alpha (PPARα), (**C**) acetyl-CoA carboxylase (ACC), and (**D**) 5′ AMP-activated protein kinase (AMPK). H9c2 cardiomyocytes were exposed to 33 mM glucose (HG) for 48 h before treatment with a combination of ASP and PPAG, as well as MET, at a dose 1 μM for 6 h. Results are expressed as the mean ± SEM of three independent experiments relative to the normal glucose (NG) control (5.5 mM). * *p* < 0.05, *** *p* < 0.001 versus NG control; # *p* < 0.05, ### *p* < 0.001 versus HG control.

Consistent with alterations in myocardial substrate metabolism, high glucose exposed H9c2 cardiomyocytes demonstrated significantly reduced mRNA expression of GLUT4 (0.71 fold; *p* ˂ 0.05) and PPARα (0.15 fold; *p* ˂ 0.001) when compared to cells under normoglycemic conditions (Figure 3A,B). This effect was concomitant to downregulated mRNA expression of ACC (0.6 fold; *p* ˂ 0.001) and upregulated AMPK levels (1.3 fold; *p* ˂ 0.05) (Figure 3C,D). The combination of aspalathin and PPAG showed an augmented effect in terms of enhancing GLUT4 levels versus metformin (0.5 vs. 1.1 fold; *p* ˂ 0.05), while displaying a similar outcome for enhancing PPARα expression (0.76 and 0.93 fold; *p* ˂ 0.001) (Figure 3A,B). Although no significant effects were observed for AMPK expression, the combination of aspalathin and PPAG could markedly increase ACC expression (*p* ˂ 0.05) (Figure 3C,D). The results on enhanced effect of the combination therapy are consistent with previous findings showing an additive outcome when metformin is combined with aspalathin than its use as a monotherapy in modulating some of the genes involved in glucose metabolism like GLUT4 in cardiomyocytes exposed to hyperglycemic conditions [18].

### 3.4. The Combination Effect of Aspalathin and PPAG on Oxidative Stress Markers

**Figure 4:**
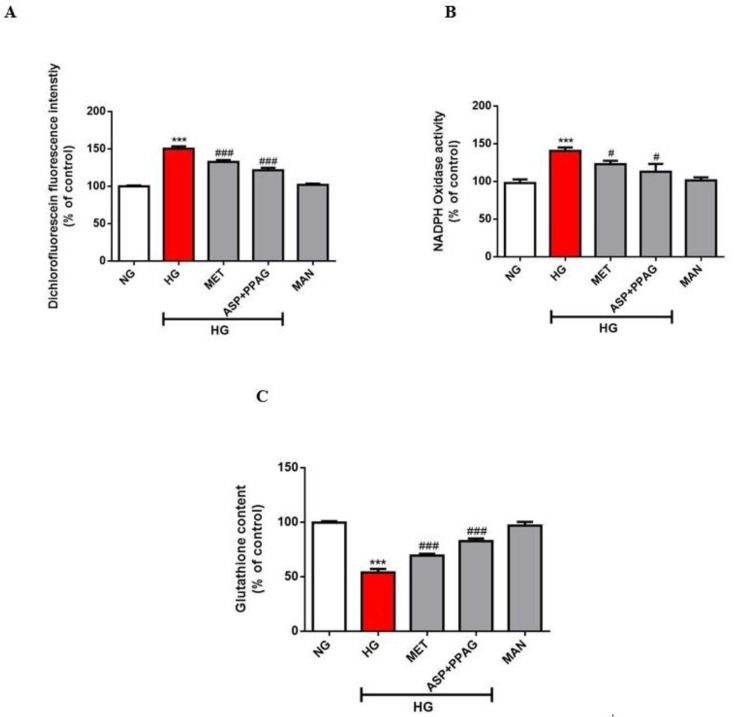
The combination effect of aspalathin (ASP) and phenylpyruvic acid-2-*O*-β-d-glucoside (PPAG) in comparison to metformin (MET) on ameliorating oxidative stress markers. This was done by measuring dichlorofluorescein intensity (**A**) to estimate productions of reactive oxygen species, NADPH oxidase activity (**B**) and glutathione content (**C**). H9c2 cardiomyocytes were exposed to 33 mM glucose (HG) for 48 h before treatment with a combination of ASP and PPAG, as well as MET, at a dose 1 μM for 6 h. Mannitol (MAN) at a concentration of 33 mM was used to rule out the effect of osmolarity. Results are expressed as the mean ± SEM of three independent experiments relative to the normal glucose (NG) control (5.5 mM). *** *p* < 0.001 versus NG control; # *p* < 0.05, ### *p* < 0.001 versus HG control.

It is generally accepted that excess ROS production through various sources including Nox, together with the depletion of intracellular antioxidants such as GSH, are implicated in hyperglycemia-induced cardiac damage. Here, exposure of H9c2 cardiomyocytes to chronic glucose concentrations significantly elevated ROS (150%; *p* ˂ 0.001) and Nox (140%; *p* ˂ 0.001) levels whilst concomitantly depleting GSH content (54%; *p* ˂ 0.001) (Figure 4A–C). The combination of aspalathin and PPAG reduced excess ROS (122%; *p* ˂ 0.001) and Nox (115%; *p* ˂ 0.05) while similarly enhancing total glutathione levels (93%; *p* ˂ 0.001) (Figure 4A–C). The combination effect of aspalathin and PPAG showed comparable efficacy to metformin in modulating markers of oxidative stress. However, these findings support the synergistic effect of aspalathin and PPAG in ameliorating oxidative stress, since PPAG has already been shown to fail in protecting against this consequence in cardiomyocytes exposed to hyperglycemic conditions [19].

### 3.5. The Combination Effect of Aspalathin and PPAG on Ameliorating DNA Damage

**Figure 5:**
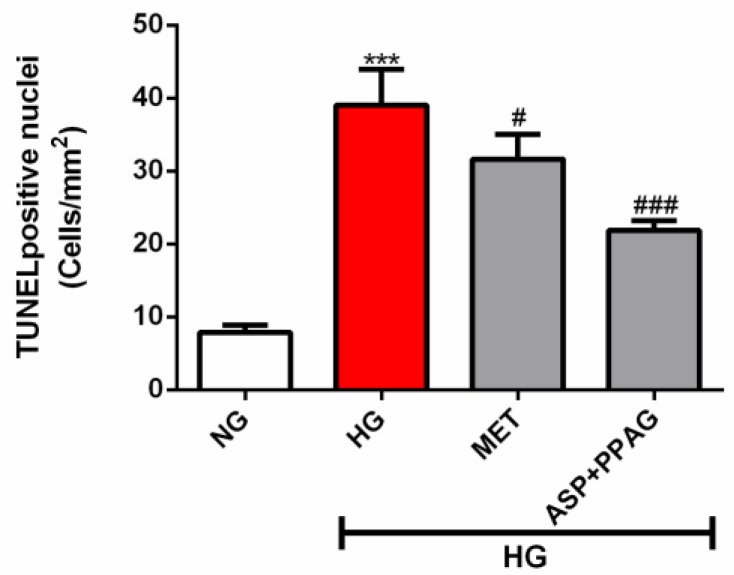
The additive effect of aspalathin (ASP) and phenylpyruvic acid-2-*O*-β-d-glucoside (PPAG) in comparison to metformin (MET) on protecting against DNA damage. H9c2 cardiomyocytes were exposed to 33 mM glucose (HG) for 48 h before treatment with a combination of ASP and PPAG, as well as MET, at a dose 1 μM for 6 h. Results are expressed as the mean ± SEM of three independent experiments relative to the normal glucose (NG) control (5.5 mM). *** *p* < 0.001 versus NG control; # *p* < 0.05, ### *p* < 0.001 versus HG control.

The TUNEL assay was used to measure DNA damage. Chronic glucose exposure significantly accelerated DNA damage (39 TUNEL positive nuclei; *p* ˂ 0.001) in H9c2 cardiomyocytes versus normoglycemic controls (Figure 5). However, treatment with the combination of rooibos compounds (22 TUNEL positive nuclei; *p* ˂ 0.001) ameliorated this and to a better extent than metformin (26 TUNEL positive nuclei; *p* ˂ 0.05) (Figure 5). These results suggest that the combination effect may be effective in promoting cell survival as evident by a reduced number of cardiomyocytes with DNA damage when compared to those exposed to high glucose only or treated with metformin. Certainly, the synergistic effects of these compounds are evident as they outperformed metformin in protecting against DNA damage. Alternatively, the use of aspalathin or PPAG as a monotherapy shows comparable results to this accomplished glucose-lowering drug in cardiomyocytes exposed to hyperglycemic conditions [18,19].

## 4. Discussion

A diabetic state is characterized by multiple metabolic dysregulations that accelerate susceptibility of the myocardium to oxidative damage [5,6]. In fact, chronic hyperglycemia during diabetes is a major phenomenon implicated in the aggravation of ROS-mediated myocardial damage [5,6]. Although commonly used anti-diabetic therapies such as insulin and metformin can control hyperglycemia [29,30], their limited efficacy in protecting the myocardium necessitates further investigations into alternative remedies. Bioactive compounds, including those found in rooibos such as aspalathin and PPAG, are already presenting diverse metabolic benefits [11,19,20,21]. However, limited data are available regarding the efficacy of combination use of such compounds versus commonly used anti-diabetic drugs like metformin. Thus, using an established in vitro model of high glucose-induced cardiac damage [17,18,19], the current study explored the combination effect induced by some of the most active compounds in rooibos (aspalathin and PPAG) in protecting against hyperglycemia-induced oxidative damage.

It is now well-accepted that myocardial metabolic inflexibility during the diabetic state is one of the earliest signs of cardiac dysfunction [5,6]. As expected, this study demonstrated that exposure of cardiomyocytes to chronic high glucose concentrations impaired myocardial metabolic flexibility, causing exacerbated FFA oxidation together with lowered glucose oxidation. In parallel, reduced GLUT4 mRNA expression could explain the suppressed glucose transport and utilization, whereas enhanced levels of AMPK corresponded with upregulated β-oxidation in these H9c2 cells. Although each molecule of glucose generates much lower ATP versus FFAs, the former is known to provide improved cardiac energy efficiency under stressful conditions [31]. AMPK is the master regulator of energy metabolism while its aberrant activation and interaction with ACCβ or also called ACC2 can prompt increased mitochondrial entry of FFAs for β-oxidation, leading to alterations in oxidative phosphorylation process [32]. As demonstrated elsewhere [18,25,33], exposing cardiomyocytes to high glucose concentrations also altered mitochondrial membrane potential in association with inducing excess ROS production that occurred together with depletion of intracellular GSH levels. Generation of oxidative stress as a result of depleted antioxidant systems could also explain accelerated DNA damage in these cardiomyocytes under hyperglycemic conditions. Thus, many therapeutic compounds are actively screened for not only modulating myocardial substrate metabolism, but for their capacity to improve intracellular antioxidants to protect against oxidative stress cellular damage [25,34].

Available evidence already shows that various extracts of rooibos can mitigate metabolic complications associated with the development of diabetes by enhancing intracellular antioxidants and modulating energy metabolism [9,35,36,37]. By activating AMPK, these extracts can control substrate metabolism including uptake and utilization of FFAs and glucose in different cell types, and this can lead to the reversal of insulin resistance and other metabolic complications [37]. In relation to the diabetic heart, the use of aspalathin and PPAG can independently modulate substrate metabolism and maintain mitochondrial membrane potential leading to attenuation of hyperglycemia-induced oxidative damage [18,19]. Although the current results did not show any significant changes for AMPK expression after exposure to metformin or combination treatment, modifications observed with ACC levels suggests that perhaps analyzing protein expression is a better method to assess the implication of AMPK in these conditions, as previously demonstrated [18]. However, an interesting finding was the capacity of the combination treatment, performing like metformin in upregulating PPARα levels under hyperglycemic conditions. PPARα plays a major role in the regulation of glucose and lipid metabolism, including mitochondrial dynamics in the diabetic heart [38], and to our knowledge, this is the first study to implicate the modulatory effect of rooibos compounds of this transcriptional factor in the myocardium. However, more studies are required to confirm the involvement of rooibos or its bioactive compounds in modulating these transcriptional factors to control energy metabolism within a diabetic heart.

Besides modulating myocardial substrate metabolism, enhancement of nuclear factor erythroid 2-related factor 2 (Nrf2) and its downstream target genes such as GSH is another mechanism implicated in the protective effect of rooibos or its bioactive compounds such as aspalathin against oxidative stress-induced damage [17,39,40]. Although PPAG lacks antioxidant-enhancing properties [19], the current findings showed that its combination with aspalathin can improve this aspect and protect against oxidative damage. In this case, the strong antioxidant and glucose-lowering properties by aspalathin [17,18] appear to be essential in complementing the bioactivity of PPAG. This could also explain our recent findings demonstrating an enhanced effect by aspalathin to improve the antidiabetic properties of metformin than the use of this glucose-lowering drug as a monotherapy in *db/db* mice [41]. Additionally, of interest in the current study was the pronounced effect, even better than that of metformin, of the combination therapy in ameliorating hyperglycemia-induced DNA damage. This may suggest synergistic effect of both compounds in preventing cellular injury may be true. This result could be related the capacity of both compounds to block BCL2-associated X protein (BAX), as previously demonstrated [18,19]. However, it is clear that the regulatory effect of these compounds against diverse molecular mechanisms of apoptosis needs further investigation. In fact, although providing important evidence regarding the possible combination effects that could be explored to understand the efficacy of rooibos compounds in protecting against hyperglycemia-induced cardiac damage, the current study is not without limitations. Firstly, the impact of treatment on lipid peroxidation as a measurement of oxidative stress is required, while the expression of genes involved in energy metabolism should still be confirmed at the protein level. Secondly, the mechanistic studies that focus on the effect of treatment in regulating cell survival mechanisms such as antioxidant response pathways like Nrf2 and related targets are still necessary. Lastly, in addition to further assessing the combined use of these compounds with glucose-lowering drugs at different doses, in vivo studies should be performed to gain an improved understanding regarding their bioavailability profile and long-term protective effect within the diabetic context.

## 5. Conclusions

Although both aspalathin and PPAG have been found to independently ameliorate hyperglycemia-associated complications, this study provides novel evidence for their cardioprotective effect when used in combination. The primary findings showed, that in addition to regulating cardiac substrate metabolism through improvement of FFA and glucose oxidation, the combination treatment could effectively reduce oxidative stress by suppressing ROS and Nox activity, while concomitantly strengthening intracellular antioxidants such as GSH. Of particular interest, the current findings suggest the pleiotropic effects of aspalathin and PPAG may contribute to its enhanced capacity to protect against hyperglycemia-induced cardiac damage.

## Data Availability

All data used to support the findings of this study are included within the article. Raw data can be available on request after publication.
